# Magnetic Resonance Imaging of Bone Marrow Cell-Mediated Interleukin-10 Gene Therapy of Atherosclerosis

**DOI:** 10.1371/journal.pone.0024529

**Published:** 2011-09-07

**Authors:** Jihong Sun, Xubin Li, Hongqing Feng, Huidong Gu, Tiffany Blair, Jiakai Li, Stephanie Soriano, Yanfeng Meng, Feng Zhang, Qinghua Feng, Xiaoming Yang

**Affiliations:** 1 Department of Radiology, Sir Run Run Shaw Hospital, Zhejiang University School of Medicine, Hangzhou, Zhejiang, China; 2 Image-Guided Bio-Molecular Interventions Section, Department of Radiology, University of Washington School of Medicine, Seattle, Washington, United States of America; 3 Department of Pathology, University of Washington School of Medicine, Seattle, Washington, United States of America; Northwestern University, United States of America

## Abstract

**Background:**

A characteristic feature of atherosclerosis is its diffuse involvement of arteries across the entire human body. Bone marrow cells (BMC) can be simultaneously transferred with therapeutic genes and magnetic resonance (MR) contrast agents prior to their transplantation. Via systemic transplantation, these dual-transferred BMCs can circulate through the entire body and thus function as vehicles to carry genes/contrast agents to multiple atherosclerosis. This study was to evaluate the feasibility of using in vivo MR imaging (MRI) to monitor BMC-mediated interleukin-10 (IL-10) gene therapy of atherosclerosis.

**Methodology:**

For *in vitro* confirmation, donor mouse BMCs were transduced by IL-10/lentivirus, and then labeled with a T2-MR contrast agent (Feridex). For *in vivo* validation, atherosclerotic apoE^−/−^ mice were intravenously transplanted with IL-10/Feridex-BMCs (Group I, n = 5) and Feridex-BMCs (Group II, n = 5), compared to controls without BMC transplantation (Group III, n = 5). The cell migration to aortic atherosclerotic lesions was monitored *in vivo* using 3.0T MRI with subsequent histology correlation. To evaluate the therapeutic effect of BMC-mediated IL-10 gene therapy, we statistically compared the normalized wall indexes (NWI) of ascending aortas amongst different mouse groups with various treatments.

**Principal Findings:**

Of *in vitro* experiments, simultaneous IL-10 transduction and Feridex labeling of BMCs were successfully achieved, with high cell viability and cell labeling efficiency, as well as IL-10 expression efficiency (≥90%). Of *in vivo* experiments, MRI of animal groups I and II showed signal voids within the aortic walls due to Feridex-created artifacts from the migrated BMCs in the atherosclerotic plaques, which were confirmed by histology. Histological quantification showed that the mean NWI of group I was significantly lower than those of group II and group III (*P*<0.05).

**Conclusion:**

This study has confirmed the possibility of using MRI to track, in vivo, IL-10/Feridex-BMCs recruited to atherosclerotic lesions, where IL-10 genes function to prevent the progression of atherosclerosis.

## Introduction

Atherosclerotic cardiovascular disease remains the leading cause of death in developed countries. A characteristic feature of atherosclerotic cardiovascular disease is its diffuse involvement of arteries across the entire human body, with the presence of multiple atherosclerotic lesions. Endovascular interventional procedures, such as balloon angioplasty and stenting, are currently used as routine “local” treatments of atherosclerotic arteries. However, these interventional approaches do not treat multiple and diffuse atherosclerosis. Thus, it is essential to seek alternatives, to meet the need of simultaneously treating all atherosclerotic arteries at once.

Recent studies have confirmed that bone marrow (BM)-derived stem-progenitor cells (SPC) can give rise to vascular progenitor cells that migrate or home to atherosclerotic arteries and differentiate into either smooth muscle cells (SMC) or endothelial cells (EC) [1]–[4]. Intravenously-transfused hematopoietic SPCs circulate in the blood system, flow through the entire body, and thereby can reach all atherosclerotic arteries. Thus, the characteristic of circulating SPCs with wide dissemination through the entire body may provide an appropriate approach for cell-mediated therapy of multiple and diffuse atherosclerosis.

Gene therapy is an exciting frontier in cardiovascular medicine [5]–[7]. Recent studies have confirmed that one of the anti-inflammatory genes, interleukin-10 (IL-10), is a crucial protective factor against the progression of atherosclerotic disease [8]–[10]. The transfer of IL-10 genes into hematopoietic SPCs prior to their transplantation to the body may allow for the simultaneous treatment of diffuse and multiple atherosclerotic lesions through the SPC-mediated, plaque-specific delivery of IL-10 genes.

Recent efforts using magnetic resonance imaging (MRI) to serially track cell transplantation have focused on labeling stem cells with MRI-detectable contrast agents, such as super-paramagnetic iron oxide agents [11]. Once magnetically labeled, stem cells may carry MR contrast agents specifically to the targets and thus be visualized under MRI [12]–[14]. The two concepts, that (a) hematopoietic SPCs can be simultaneously transferred with therapeutic genes and MR contrast agents prior to their transplantation; and (b) the dual-transferred hematopoietic SPCs can circulate through the body and thus function as vehicles to carry genes/contrast agents to the atherosclerotic plaques, motivated us to develop a plaque-specific MRI technique, to monitor SPC-mediated vascular gene therapy. The aim of this study was to confirm the possibility of using in vivo MRI to monitor IL-10 gene-transduced, MR contrast agent-labeled bone marrow cells (BMC) that were recruited to atherosclerosis for preventing the progression of atherosclerotic disease.

## Materials and Methods

### Study design

This study was divided into three phases. Phase I included a series of *in vitro* experiments to confirm the feasibility of simultaneous IL-10 gene transduction and Feridex labeling of BMCs, with determination of the cell viability and cell labeling efficiency, as well as IL-10 gene expression efficiency. Phase II included a series of *in vivo* experiments to validate the feasibility of using MRI to track the gene/contrast agent dual-modified BMCs migrated to atherosclerotic lesions, which was confirmed by subsequent MR-histology correlation. Phase III focused on histologic evaluation of the role of BMC-mediated IL-10 gene therapy in preventing the progression of atherosclerotic plaques. The animal protocol for this study was approved by University of Washington's Institutional Animal Care and Use Committee (Protocol Number: 4120-01) and complied with National Institutes of Health guidelines [15].

### In Vitro Experiments

#### MIL-10-lentivirus production

**Figure 1:**
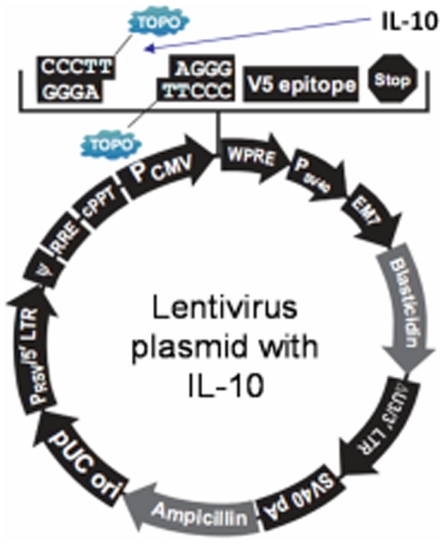
Schematic diagram of construction of IL-10/Lentivirus. IL-10/lentivirus-based plasmid was made by inserting the mIL-10 cDNA into the pLenti6.3/V5-TOPO vector.

A lentivirus-based plasmid was made by inserting the mIL-10 cDNA into the pLenti6.3/V5-TOPO vector (Invitrogen, Carlsbad, CA, USA) (Fig. 1). This pLenti-mIL-10 plasmid was then used to transfect 293FT cells with a Packaging Mix (pLP1, pLP2 and pLP-VSVG, Invitrogen) to produce mIL-10 lentivirus.

#### BMC Extraction

BMCs were extracted from seven C57/BL donor mice (six weeks old, Harlan Laboratories, Inc., Kent, WA, USA) by using the previously-established protocol [12], [13]. Briefly, the femurs and tibias of donor mice were excised. Then, the marrow was aspirated using a syringe with a 28-gauge needle and collected in a tube. After lysing red blood cells with human RBC lysis buffer (Boston Bioproducts, Worcester, MA, USA), the purified BMCs were washed with phosphate-buffered saline (PBS).

#### IL-10 transduction and Feridex-labeling of BMCs

According to the manufacturer's protocol (Invitrogen, Carlsbad, CA, USA), we first transduced the donor BMCs with lentiviral vectors that carried the IL-10 genes. The transduction of BMCs was performed at multiplicity of infection = 1, using several agents of QBSF 58 serum-free medium (Quality Biological Inc. Gaithersburg, MD, USA), 8-ug/ml polybrene (Sigma, St. Louis, MO, USA), 50-ug/ml mSCF, 50-ug/ml hFlt3, and 20-ug/ml hTPO (PeproTech Inc. Rocky Hill, NJ, USA) for 48 hours. After transduction, the IL-10-BMCs were labeled with a superparamagnetic iron oxide MR contrast agent (25-ug Fe/ml, Feridex I.V.; Berlex Laboratories, Inc., Wayne, NJ, USA) and a poly-cation transduction agent (375 ng/ml, Poly-L-lysine; Sigma, St. Louis, MO, USA). We also set different control groups, including (i) Feridex-labeled BMCs; (ii) IL-10-transduced BMCs; and (iii) unmodified BMCs. The *in vitro* experiments for each of the cell groups were performed in triplicate.

#### Laboratory Confirmation

Successful simultaneous transduction and labeling of BMCs were confirmed by immunohistochemical (IHC) staining for IL-10 gene expression and Prussian blue staining for Feridex labeling. IHC was performed with a primary goat monoclonal antibody against mouse IL-10 (Sigma, St. Louis, MO, USA) using an anti-goat HPR-DAB cell & tissue staining kit according to the manufacturer's specification (R&D Systems, Inc., Minneapolis, MN, USA). Cells were then counterstained with hematoxylin (Ricca Chemical Company, Arlington, Texas, USA). Prussian blue staining was performed by incubating paraformaldehyde-fixed, Feridex–labeled BMCs with 2% potassium ferrocyanide in 2% hydrochloric acid for 30 minutes. Cells were then counterstained with eosin (Sigma Aldrich Inc., St. Louis, MO, USA).

#### Evaluation of Cell Viability and Labeling Efficiency

Viability of the transduced and labeled cells was determined by a trypan blue exclusion assay with subsequent cell counting. The cell labeling/gene transduction efficiencies were determined by percentages of Feridex-positive cells (as intracellular blue-colored dots with Prussian blue staining) and IL-10 positive cells (as intracellular brown-colored precipitates with IHC), which were counted in three fields of each cell study group under a microscope (Olympus BX51, Tokyo, Japan).

### *In Vivo* Validation

#### Animal Study Groups and Cell Transplantation

**Figure 2:**
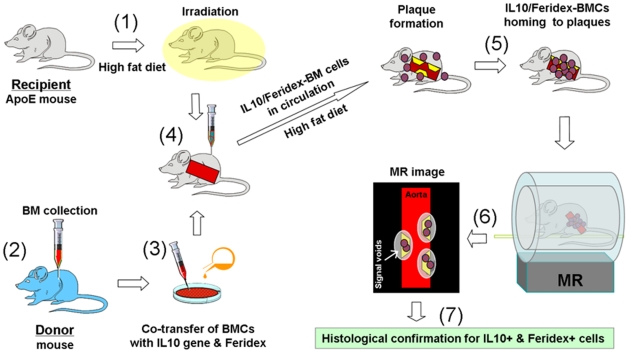
Schematic diagram of the experimental design and steps for the *in vivo* studies. These studies include (1) creating atherosclerotic plaques in the ascending aorta of the recipient apoE^−/−^ mouse by feeding a high fat diet; (2) extracting bone marrow cells (BMCs) from the donor mouse; (3) co-transferring the donor BMCs with IL-10 gene and Feridex *in vitro*; (4) transplanting IL-10/Feridex-BMCs to the recipient atherosclerotic mouse, which is irradiated prior to transplantation; (5) IL-10/Feridex-BMCs homing to aortic atherosclerotic plaques; (6) in vivo MRI detecting Feridex-created signal voids from the recruited IL-10/Feridex BMCs in atherosclerotic plaques; and (7) confirming the successful recruitment of co-transferred cells to the plaques by histology correlation.

Figure 2 summarizes the experimental design and steps of the *in vivo* validation study. Fifteen recipient atherosclerotic apolipoprotein E knockout (apoE^−/−^) mice (8 weeks old, B6.129P2; Jackson Laboratory, Bar Harbor, Maine, USA) were divided into three study groups: group I with IL-10/Feridex BMC transplantation (n = 5), group II with Feridex-labeled BMC transplantation (n = 5), and group III with no cell transplantation to serve as a control (n = 5). All animals were fed a high fat diet (15% butter and 2% cholesterol, RD Western Diet; Research Diets, Inc., New Brunswick, NJ, USA) for approximately three months [16].

Before BMC transplantations, ten recipient apoE^−/−^ mice in groups I and II were irradiated with a dose of 900 rads. Then, the IL-10/Feridex BMCs or Feridex-labeled BMCs were transplanted into the recipient mice via a tail vein injection. Each recipient mouse received approximately 1×10^7^ cells suspended in 0.3 to 0.4 mL PBS. After cell transplantations, all animals were fed the same high fat diet for additional four weeks.

#### In Vivo MRI

Approximately four weeks later, the aortic atherosclerotic lesions of these apoE^−/−^ mice were examined using a 3T MRI scanner (Achieva, Philips Healthcare, Best, The Netherlands). The mice were placed in a prone position within a Philips solenoid mouse-specific coil (Philips Research, Hamburg, Germany). Electrocardiogram (ECG)-gating (Model 1025 Monitoring & Gating System, SA Instruments, Inc., Stony Brook, NY, USA) was performed via attaching two ECG needles to the right-upper and left-lower extremities of the mouse. Axial T2-weighted and proton density (PD)-weighted black blood MR images of the ascending aorta were obtained during the ventricular diastole by using a turbo spin-echo (TSE) sequence, with T2-MRI parameters of repetition time (TR) at 4 heart beating intervals, 60-ms echo time (TE), 40×40-mm field of view (FOV), 9 TSE factor, 1-mm slice thickness with no slice gap, 168×162 matrix, and 8 signal average (NSA). PD-weighted MRI parameters included TR at 4 heart beating intervals, 10-ms TE, 40×40-mm FOV, 3 TSE factor, 1-mm slice thickness without slice gap, 168×162 matrix, and 8 NSA.

#### Histologic Confirmation and Correlation

After achieving satisfactory MR images, the animal was anesthetized by intraperitoneal injection of 100–150 uL pentobarbital (0.25–0.38 mg/g) and then exsanguinated by endovascular perfusion with 4% paraformaldehyde via an open-chest, left cardiac ventricle puncture approach. The ascending aorta, approximately 5 mm in length measured from the cardiac base, was harvested, and then serially cryosectioned at 6-µm slice thickness. Then, we stained the slides with (a) Prussian blue staining for confirming Feridex-positive cells; and (b) immunofluorescent dual staining for detecting dextran shells of Feridex particles and IL-10 gene expression (Abcam, Cambridge, MA, USA) in the atherosclerotic plaques. In addition, DAPI (Vector Laboratories, Inc., Burlingame, CA, USA) was used for nuclei staining. A previous study had extensively evaluated the phenotypes of migrated BMCs localized in the atherosclerosis of the same apoE^−/−^ mouse model, and found them to primarily give rise to smooth muscle cells [1]. Thus, in this study, we did not attempt to determine if BMCs remained in undifferentiated form or followed downstream differentiation pathways.

To evaluate the therapeutic effect of BMC-mediated IL-10 gene therapy on preventing the progression of atherosclerotic plaques, we used ImageJ (1.43 u, National Institutes of Health, Bethesda, MD, USA) to quantitatively measure a normalized wall index (NWI) of ascending aorta of each recipient apoE^−/−^ mouse. NWI was calculated by dividing the aortic wall area by the total aortic area at cross-sectional views of digitized microscopic images [17]. The NWI provided a quantitative measure of atherosclerotic plaque severity. The lower the NWI value is, the less severe the atherosclerotic plaque is.

#### Statistical Analysis

Statistical analysis was blindly performed by two investigators using SPSS 13.0 for Windows (SPSS Inc., Chicago, IL, USA). A one-way ANOVA was used to assess the significance of differences in the mean NWIs among the three mouse groups with various treatments. A *P* value of less than 0.05 was considered statistically significant.

## Results

**Figure 3:**
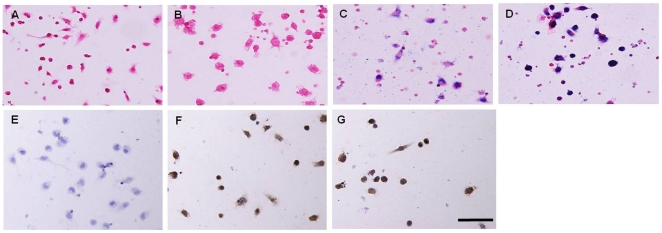
Histologic examination of the *in vitro* experiments. (A–D) Prussian blue staining of (A) unmodified BMCs, (B) IL-10-transduced BMCs, (C) Feridex-labeled BMCs, and (D) IL-10-transduced and Feridex-labeled BMCs. Feridex-positive cells represent with intracellular blue-colored dots (C and D). (E–G) Immunohistochemical staining of (E) unmodified BMCs, (F) IL-10-transduced BMCs, and (G) IL-10-transduced and Feridex-labeled BMCs. Intracellular IL-10 gene expression represents as intracellular brown-colored precipitates (F and G). 200×. Bar: 100 µm.

Of *in vitro* experiments, the viability of IL-10/Feridex-BMCs was 90%, while the viabilities for Feridex-BMCs and IL-10-BMCs were 95%, in comparison to the 98% viability of the unmodified BMCs. The efficiencies of Feridex labeling for the non-transduced and IL-10-transduced BMCs were approximately 90% and 92%, respectively. The efficiency of IL-10 gene transduction was 95%. Prussian blue staining and IHC staining confirmed the successful simultaneous IL-10 transduction and Feridex labeling of BMCs (Fig. 3).

**Figure 4:**
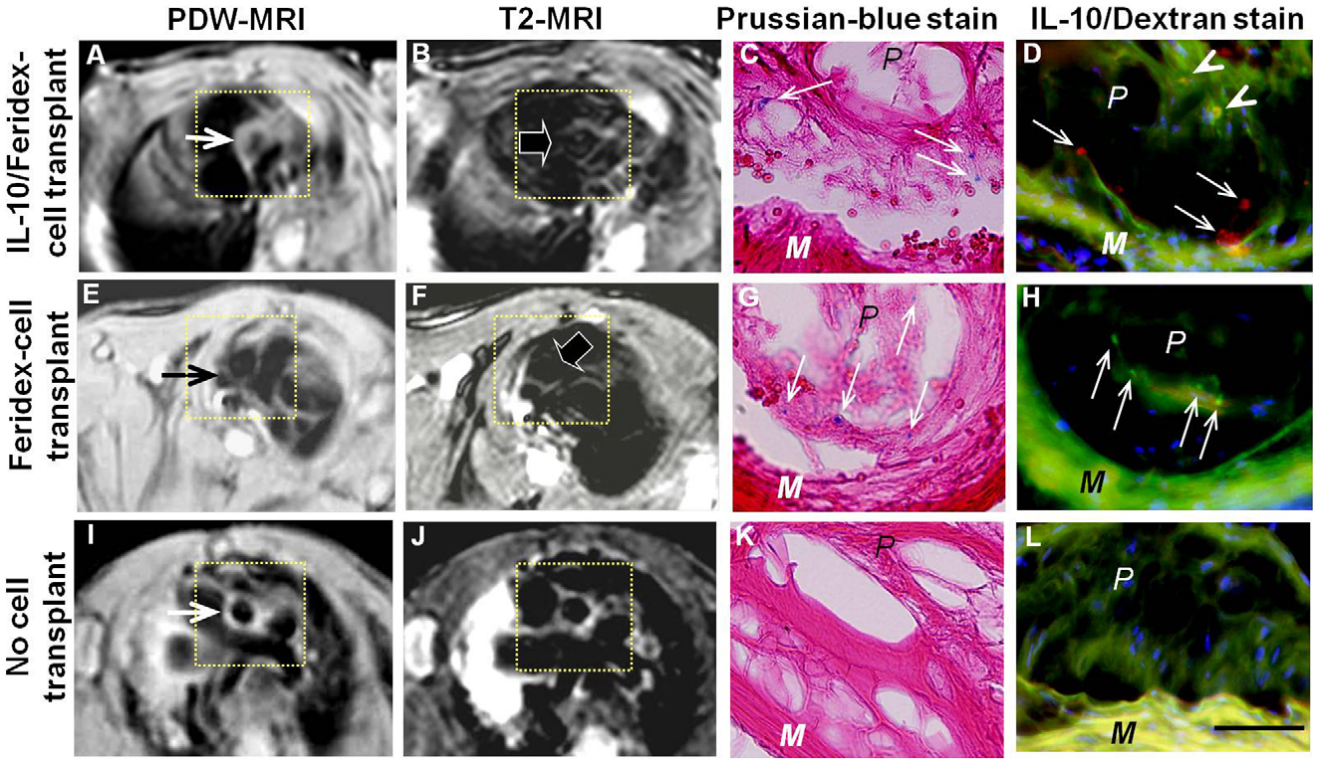
Representative MRI-histologic correlation of atherosclerotic ascending aorta (insets) of apoE^−/−^ mice. (A–D) ApoE^−/−^ mice with IL-10/Feridex-BMC transplantation. (E–H) ApoE^−/−^ mice with Feridex-BMC transplantation. (I–L) ApoE^−/−^ mice with no cell transplantation as a control. (A, E, I) Proton-density-weighted (PDW) MRI shows thickening of the aortic walls due to formation of atherosclerotic lesions (arrows on A, E, I). (B, F, J) T2-weighted MRI shows signal voids (arrows on B and F) at the aortic walls with Feridex-labeled and/or IL-10-transduced BMC transplantation, where are not seen in the aortic wall of the control group (J). (C, G, K) Prussian blue staining shows the Feridex-positive BMCs migrated to the atherosclerotic aortic walls (arrows in C and G), which are not visualized in the aortic wall of the control group (K). (D, H, L) Immunofluorescent dual staining confirms simultaneous IL-10 gene expression (as red-colored spots, arrows on D), IL-10 overlap with dextran shells of Feridex particles (as orange-colored dots, arrowheads on D) and dextran shells of Feridex particles (as green-colored dots, arrows on H), which are not seen in the aortic wall of the control group (L). 400×. Bar: 100 µm. P = atherosclerotic plaque; M = medial. Blue color indicates nuclei.

Of the *in vivo* experiments, T2-MRI of animal groups I and II demonstrated signal voids of the aortic walls due to Feridex-created artifacts from the migrated IL-10/Feridex-BMCs in atherosclerotic lesions, which were confirmed as Feridex- and/or IL-10-positive cells with immunofluorescent dual staining. These findings were not seen in the control group III (Fig. 4). Histological examination with Prussian blue staining demonstrated the formation of the atherosclerotic plaques in all 15 apoE^−/−^ mice.

**Figure 5:**
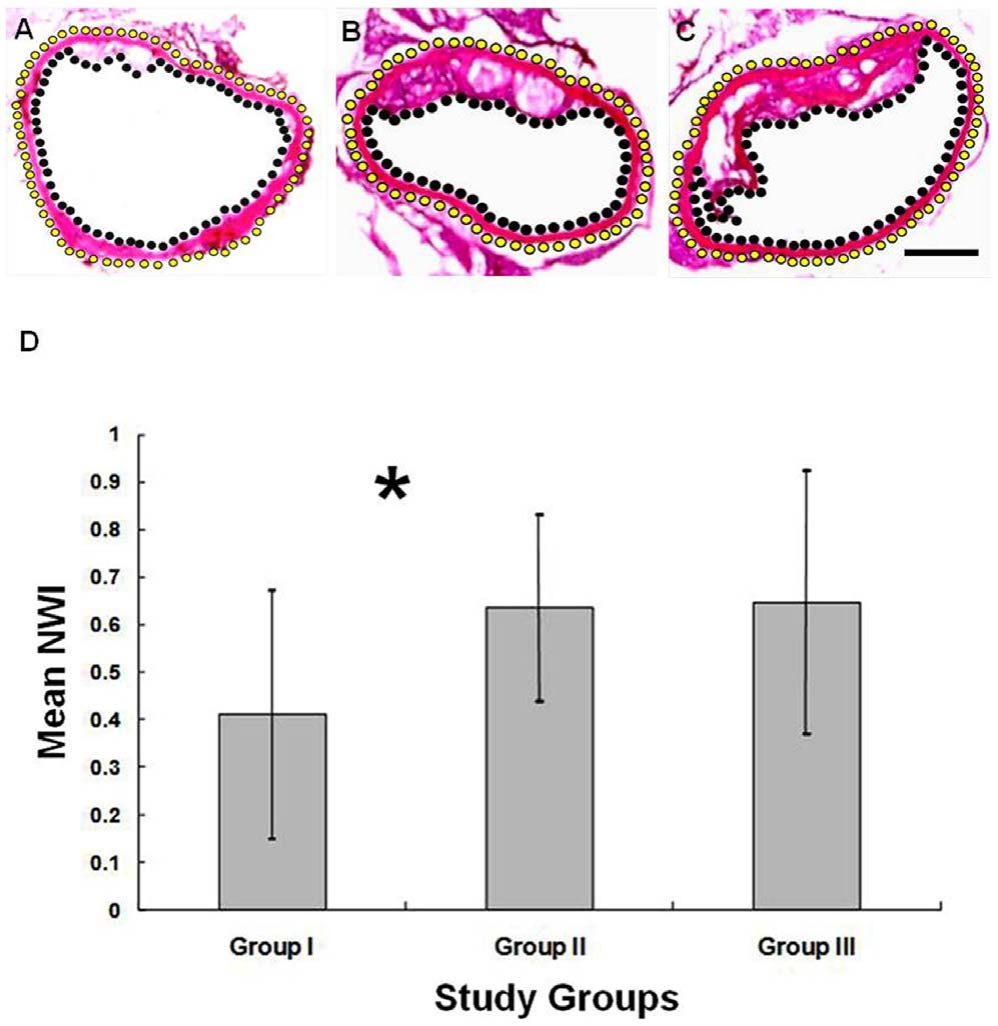
Representative histologic images of ascending aorta with measurements of normalized wall indexes (NWI) among three groups. (A) Mouse group I, transplanted with IL-10/Feridex-BMCs. (B) Group II, transplanted with Feridex-BMCs. (C) Group III with no cell transplantation. Yellow dotted lines and black dotted lines outline the measured aortic wall area. Atherosclerotic plaques are clearly seen in groups II and III (B and C). 12.5×. Bar: 200 µm. (D) Comparison of NWIs among the three groups, demonstrating that the mean NWI of group I is significantly lower than those of the control group II and group III (* *P*<0.05), with no significant difference between two control groups II and III.

Of the evaluation of BMC-mediated IL-10 gene therapy of atherosclerosis, histological quantitative measurements showed that the mean NWI of animal group I was significantly lower than those of control animal group II and group III (*P*<0.05), while there was no significant difference on the mean NWIs between the animal groups II and III (Fig. 5).

## Discussion

BMCs can differentiate into vascular cells that participate in the formation of different types of atherosclerotic plaques and transplant-associated vasculopathy [3], [4]. Intracellular MR contrast agents, such as Feridex or motexafin gadolinium, can be used to label different types of cells, so that the cells become MR-detectable [18]–[20]. Recent development of a BMC-mediated, plaque-specific MRI technique enables monitoring of BMCs trafficking to atherosclerotic lesions [12], [21]. A more recent study demonstrated the possibility of co-transferring BMCs with a green fluorescent protein (GFP) gene and an intracellular T1-MR contrast agent [21]. To the best of our knowledge, the current study represents the first attempt to validate the feasibility of using in vivo MRI to track the recruitment of BMCs, which were co-transferred with a therapeutic gene (IL-10 gene) and a T2- MR contrast agent (Feridex), into the aortic walls to aid in the prevention of atherosclerosis.

In this study, through serial in vitro experiments we first evaluated the possibility of simultaneously transducing and labeling BMCs with IL-10 genes and Feridex, which confirmed the achievements of high cell viability and labeling efficiency, as well as IL-10 expression efficiency (≥90%). Subsequently, via serial *in vivo* experiments using atherosclerotic apoE^−/−^ mouse models, we successfully validated the feasibility of using in vivo MRI to track and localize the IL-10/Feridex-BMCs that migrated to the atherosclerotic aortic walls, where T2-MRI showed signal voids of the aortic walls due to Feridex-created artifacts from the recruited Feridex-BMCs in atherosclerotic plaques. The MRI findings were confirmed by subsequent histology correlations.

One of the essential strategies in the management of atherosclerotic cardiovascular disease is developing new preventative approaches to inhibit the progress of multiple and diffuse atherosclerosis. Transplantation of SPCs expressing IL-10 genes, as reported in the current study, represents an effort for such technical development. IL-10 is a pleiotropic cytokine, which is produced primarily by macrophages and lymphocytes. Due to its anti-inflammatory and anti-apoptotic properties, IL-10 has been proven to be anti-atherogenic and atheroprotective [22], [23]. The potential anti-atherogenic mechanisms of IL-10 genes include (i) modulating the signals between two subpopulations of lymphocyte T helper cells to inhibit the production of proinflammatory cytokines [24], [25]; (ii) diminishing apoptotic activity within the plaques to decrease the formation of the necrotic cores [22], [26], [27]; (iii) inhibiting the synthesis of metalloproteinases and stimulating the production of its inhibitors to promote the stabilities of atherosclerotic plaques [10], [28]; and (iv) modulating lipid metabolisms by enhancing both uptake and efflux of cholesterol in macrophage foam cells [22], [29]. In the present study, we have initially confirmed the potential role of BMC-mediated IL-10 gene delivery in preventing the progression of atherosclerosis, with histological evidence showing that the NWI is significantly lower in the study group with transplantation of IL-10 BMCs in comparison to the control groups transplanted with Feridex-BMCs or without BMC transplantation.

Further efforts need to focus on optimizing the protocol to obtain the best time-points for cell transplantation, the peak of cell-mediated IL-10 gene expression, and MR imaging. This new approach only allows us to confirm the successful recruitment of IL-10/Feridex BMCs to atherosclerotic plaques. Further development of a method for *in vivo* estimating the functions of these transplanted gene-cells after their recruitment to atherosclerotic plaques is warranted.

In conclusion, this study has confirmed the possibility of using in *vivo* MRI to track IL-10/Feridex-BMCs migrated to atherosclerotic lesions, where IL-10 genes function to prevent or slow the progression of atherosclerosis. This technique may open a new avenue for treatment of atherosclerotic cardiovascular diseases using MR-integrated, BMC-mediated IL-10 gene therapy.

## References

[1] Xu Y, Arai H, Zhuge X, Sano H, Murayama T (2004). Role of bone marrow-derived progenitor cells in cuff-induced vascular injury in mice.. Arterioscler Thromb Vasc Biol.

[2] Orlic D, Kajstura J, Chimenti S, Bodine D, Leri A (2003). Bone marrow stem cells regenerate infarcted myocardium.. Pediatr Transplant.

[3] Krause D, Theise N, Collector M, Henegariu O, Hwang S (2001). Multi-organ, multi-lineage engraftment by a single bone marrow-derived stem cell.. Cell.

[4] Sata M, Saiura A, Kunisato A, Tojo A, Okada S (2002). Hematopoietic stem cells differentiate into vascular cells that participate in the pathogensis of atherosclerosis.. Nature Medicine.

[5] Nabel E (1995). Gene therapy for cardiovascular disease.. Circulation.

[6] Sinnaeve P, Varenne O, Collen D, Janssens S (1999). Gene therapy in the cardiovascular system: an update.. Cardiovasc Res.

[7] Yang X (2003). Imaging of vascular gene therapy.. Radiology.

[8] Namiki M, Kawashima S, Yamashita T, Ozaki M, Sakoda T (2004). Intramuscular gene transfer of interleukin-10 cDNA reduces atherosclerosis in apolipoprotein E-knockout mice.. Atherosclerosis.

[9] Pinderski Oslund LJ, Hedrick CC, Olvera T, Hagenbaugh A, Territo M (1999). Interleukin-10 blocks atherosclerotic events in vitro and in vivo.. Arterioscler Thromb Vasc Biol.

[10] Mallat Z, Besnard S, Duriez M, Deleuze V, Emmanuel F (1999). Protective role of interleukin-10 in atherosclerosis.. Circ Res.

[11] Bulte J, Douglas T, Witwer B, Zhang SC, Strable E (2001). Magnetodendrimers allow endosomal magnetic labeling and in vivo tracking of stem cells.. Nat Biotechnol.

[12] Qiu B, Gao F, Walczak P, Zhang J, Kar S (2007). In vivo MR imaging of bone marrow cells trafficking to atherosclerotic plaques.. J Magn Reson Imaging.

[13] Gao F, Kar S, Zhang J, Qiu B, Walczak P (2007). MRI of intravenously injected bone marrow cells homing to the site of injured arteries.. NMR Biomed.

[14] Qiu B, Yang X (2008). Molecular MRI of hematopoietic stem-progenitor cells: in vivo monitoring of gene therapy and atherosclerosis.. Nat Clin Pract Cardiovasc Med.

[15] National Institues of Health (revised 1985). Guide for the care and use of laboratory animals..

[16] Russell J (2003). Of mice and men, rats and atherosclerosis.. Cardiovasc Res.

[17] Tang TY, Howarth SP, Miller SR, Graves MJ, U-King-Im JM (2008). Correlation of carotid atheromatous plaque inflammation using USPIO-enhanced MR imaging with degree of luminal stenosis.. Stroke.

[18] Shapiro EM, Sharer K, Skrtic S, Koretsky AP (2006). In vivo detection of single cells by MRI.. Magn Reson Med.

[19] Bos C, Delmas Y, Desmouliere A, Koretsky AP (2004). In vivo MR imaging of intravascularly injected magnetically labeled mesenchymal stem cells in rat kidney and liver.. Radiology.

[20] Chae EY, Song EJ, Sohn JY, Kim ST, Woo CW (2010). Allogeneic renal graft rejection in a rat model: in vivo MR imaging of the homing trait of macrophages.. Radiology.

[21] Qiu B, Treuting P, Zhan X, Xie D, Frevert CW (2010). Dual transfer of GFP gene and MGd into stem-progenitor cells: toward in vivo MRI of stem cell-mediated gene therapy of atherosclerosis.. Acad Radiol.

[22] Han X, Kitamoto S, Wang H, Boisvert WA (2010). Interleukin-10 overexpression in macrophages suppresses atherosclerosis in hyperlipidemic mice.. FASEB J.

[23] Kleemann R, Zadelaar S, Kooistra T (2008). Cytokines and atherosclerosis: a comprehensive review of studies in mice.. Cardiovasc Res.

[24] Moore KW, de Waal Malefyt R, Coffman RL, O'Garra A (2001). Interleukin-10 and the interleukin-10 receptor.. Annu Rev Immunol.

[25] Blanco E, Monux G, Mas A, Serrano FJ, de la Concha EG (2008). Role of IL-10 promoter polymorphisms in the development of severe aorto-iliac occlusive disease.. Hum Immunol.

[26] Pinderski LJ, Fischbein MP, Subbanagounder G, Fishbein MC, Kubo N (2002). Overexpression of interleukin-10 by activated T lymphocytes inhibits atherosclerosis in LDL receptor-deficient Mice by altering lymphocyte and macrophage phenotypes.. Circ Res.

[27] Mallat Z, Heymes C, Ohan J, Faggin E, Leseche G (1999). Expression of interleukin-10 in advanced human atherosclerotic plaques: relation to inducible nitric oxide synthase expression and cell death.. Arterioscler Thromb Vasc Biol.

[28] Lacraz S, Nicod LP, Chicheportiche R, Welgus HG, Dayer JM (1995). IL-10 inhibits metalloproteinase and stimulates TIMP-1 production in human mononuclear phagocytes.. J Clin Invest.

[29] Rubic T, Lorenz RL (2006). Downregulated CD36 and oxLDL uptake and stimulated ABCA1/G1 and cholesterol efflux as anti-atherosclerotic mechanisms of interleukin-10.. Cardiovasc Res.

